# Crystal structure of 2-aza­niumyl-3-bromo-6-oxo-5,6-di­hydro­pyrido[1,2-*a*]quinoxalin-11-ium dibromide

**DOI:** 10.1107/S2056989014026127

**Published:** 2015-01-01

**Authors:** Md. Serajul Haque Faizi, Natalia O. Sharkina, Turganbay S. Iskenderov

**Affiliations:** a Department of Chemistry, Indian Institute of Technology Kanpur, Kanpur, UP 208 016, India; b National Taras Shevchenko University, Department of Chemistry, Volodymyrska str. 64, 01601 Kyiv, Ukraine

**Keywords:** crystal structure, bromide, pyrido[1,2-*a*]quinoxalin-11-ium, C—H⋯Br inter­actions

## Abstract

The title salt, C_12_H_10_BrN_3_O^2+^·2Br^−^, was synthesized from the reaction of *N* ^1^,*N* ^4^-bis­(pyridin-2-yl­methyl­idene)benzene-1,4-di­amine and bromine in a methanol solution. All non-H atoms of the 2-aza­niumyl-3-bromo-6-oxo-5,6-di­hydro­pyrido[1,2-*a*]quinoxalin-11-ium cation are nearly coplanar, the maximum deviation being 0.114 (4) Å. In the crystal, the cations and anions are linked through N—H⋯Br hydrogen bonds and weak C—H⋯Br inter­actions, forming a three-dimensional supra­molecular architecture. A short Br⋯Br contact [3.3088 (9) Å] is observed in the crystal.

## Related literature   

For applications of quinoxalines, see: Duffy *et al.* (2002 ▸); Gazit *et al.* (1996 ▸); Harmenberg *et al.* (1991 ▸); Naylor *et al.* (1993 ▸). For types of quinoxalines and a structure similar to title compound, see: Eiden & Peter (1966 ▸); Koner & Ray (2008 ▸); Fritsky *et al.* (2006 ▸); Kanderal *et al.* (2005 ▸); Moroz *et al.* (2012 ▸). For background to and applications of related compounds, see: Faizi & Sen (2014 ▸); Faizi *et al.* (2014 ▸).
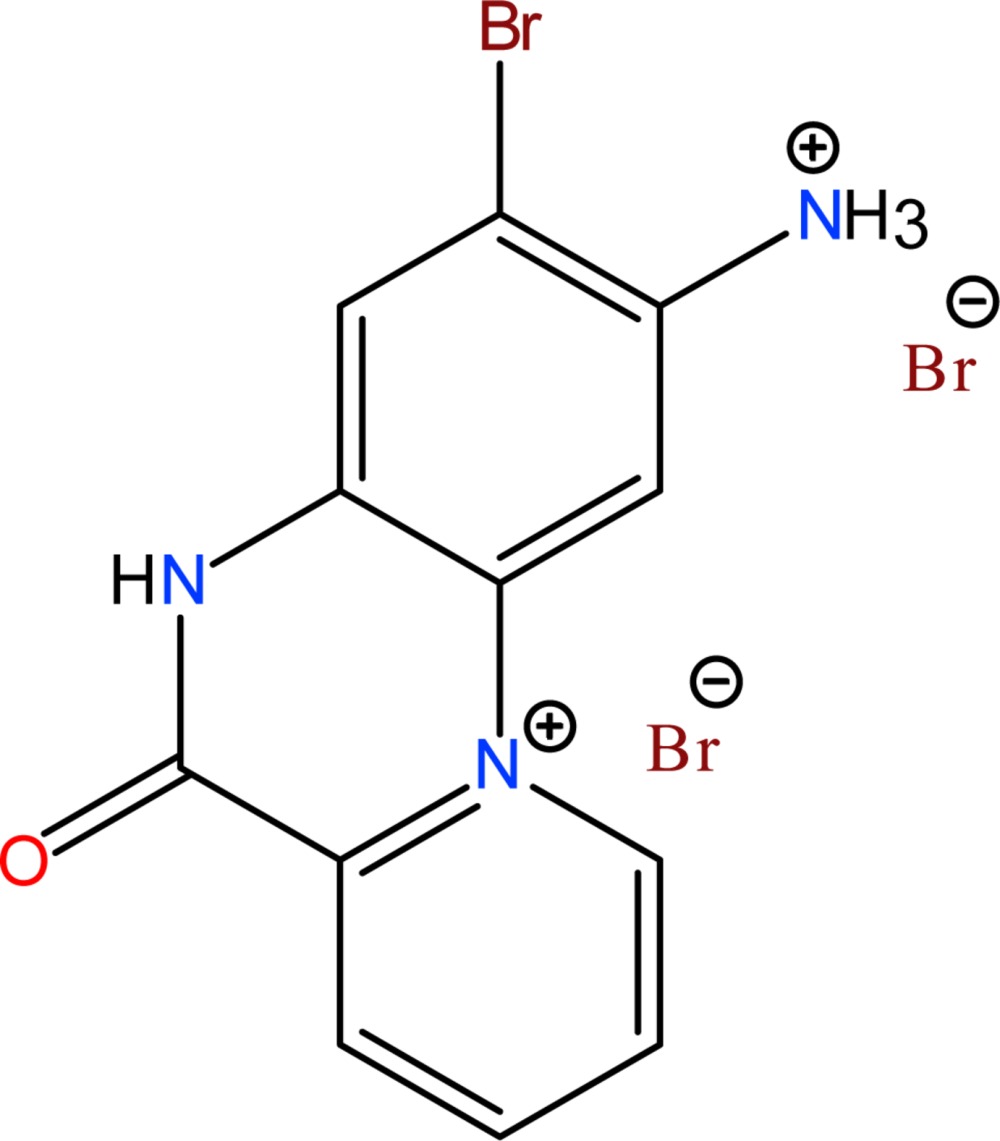


## Experimental   

### Crystal data  

C_12_H_10_BrN_3_O^2+^·2Br^−^
*M* *_r_* = 451.96
Monoclinic, 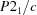

*a* = 5.6782 (2) Å
*b* = 11.9822 (4) Å
*c* = 20.2528 (7) Å
β = 90.891 (2)°
*V* = 1377.78 (8) Å^3^
*Z* = 4
Mo *K*α radiation
μ = 8.78 mm^−1^
*T* = 100 K
0.30 × 0.25 × 0.20 mm

### Data collection  

Bruker SMART APEX CCD diffractometer
Absorption correction: multi-scan (*SADABS*; Bruker, 2001 ▸) *T*_min_ = 0.178, *T*_max_ = 0.273
14369 measured reflections
2424 independent reflections
1804 reflections with *I* > 2σ(*I*)
*R*_int_ = 0.106

### Refinement  

*R*[*F* ^2^ > 2σ(*F* ^2^)] = 0.047
*wR*(*F* ^2^) = 0.130
*S* = 0.97
2424 reflections
173 parameters
H-atom parameters constrained
Δρ_max_ = 1.23 e Å^−3^
Δρ_min_ = −0.88 e Å^−3^

### 

Data collection: *SMART* (Bruker, 2003 ▸); cell refinement: *SAINT* (Bruker, 2003 ▸); data reduction: *SAINT*; program(s) used to solve structure: *SIR97* (Altomare *et al.*, 1999 ▸); program(s) used to refine structure: *SHELXL97* (Sheldrick, 2008 ▸); molecular graphics: *DIAMOND* (Brandenberg & Putz, 2006 ▸); software used to prepare material for publication: *DIAMOND*.

## Supplementary Material

Crystal structure: contains datablock(s) global, I. DOI: 10.1107/S2056989014026127/xu5829sup1.cif

Structure factors: contains datablock(s) I. DOI: 10.1107/S2056989014026127/xu5829Isup2.hkl

Supporting information file. DOI: 10.1107/S2056989014026127/xu5829Isup3.cml

**Fig. 1.**
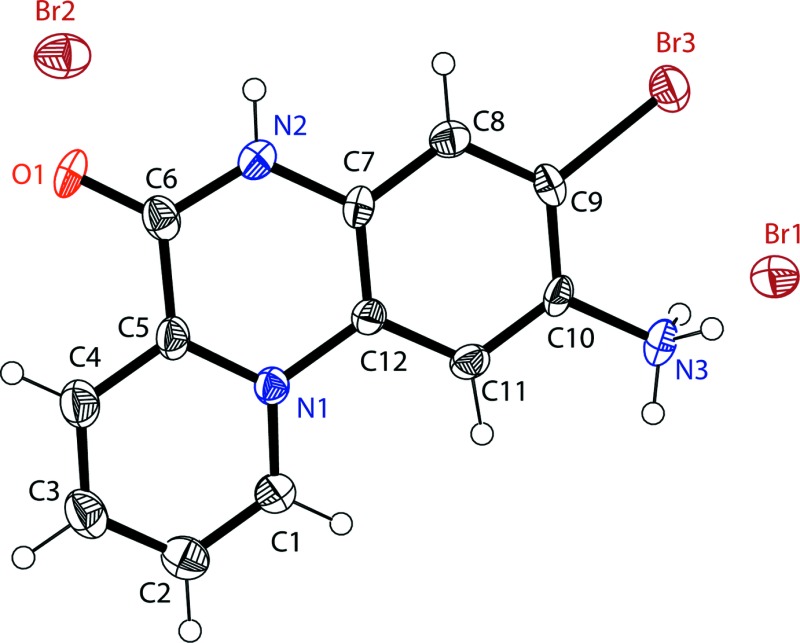
The molecular conformation and atom-numbering scheme for the title compound, with non-H atoms drawn as 40% probability displacement ellipsoids.

. DOI: 10.1107/S2056989014026127/xu5829fig1.tif
The mol­ecular conformation and atom-numbering scheme for the title compound, with non-H atoms drawn as 40% probability displacement ellipsoids.

CCDC reference: 1036569

Additional supporting information:  crystallographic information; 3D view; checkCIF report

## Figures and Tables

**Table 1:** Hydrogen-bond geometry (, )

*D*H*A*	*D*H	H*A*	*D* *A*	*D*H*A*
N2H2*A*Br1^i^	0.88	2.46	3.322(5)	167
N3H1*N*3Br2^ii^	0.91	2.53	3.432(5)	171
N3H2*N*3Br2^iii^	0.91	2.42	3.287(5)	160
N3H3*N*3Br1	0.91	2.50	3.374(5)	162
C2H2Br2^iv^	0.95	2.91	3.813(6)	160
C3H3Br1^v^	0.95	2.85	3.752(7)	160
C8H8Br1^i^	0.95	2.86	3.672(6)	144
C11H11Br2^ii^	0.95	2.80	3.639(6)	148

Symmetry codes: (i) 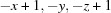; (ii) 
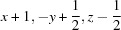
; (iii) 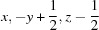; (iv) 
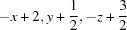
; (v) 
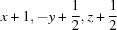
.

## supplementary crystallographic information

### S1. Comment

Quinoxalines are an important class of heterocyclic compounds, some of which are found to be useful as fluorophores, dyes, and antibiotics (Duffy *et al.*, 2002; Gazit *et al.*, 1996). Many drug candidates bearing quinoxaline core structures are in clinical trials in antiviral, anticancer and *CNS* (central nervous system) therapeutic areas (Harmenberg *et al.*, 1991; Naylor *et al.*, 1993). The present work is a part of an ongoing structural study of Schiff bases and their utilization in the synthesis of previously unknown organic and polynuclear coordination compounds (Faizi & Sen, 2014; Faizi *et al.*, 2014; Moroz *et al.*, 2012), and we report here synthesis and structure of 2-azaniumyl-3-bromo-6-oxo-5,6-dihydropyrido[1,2-*a*]quinoxalin-11-ium bromide (ABODQ). There are very few examples similar to title compound have been reported in the literature (Eiden & Peter, 1966).

The title compound was synthesized from the reaction of two equimolar amounts of molecular bromine and pyridine derivative Schiff base *N1*,*N4*-bis(pyridine-2-ylmethylene) benzene-1,4-diamine (BPYBD). The cyclization occurs by oxidation of BPYBD, reduction of molecular bromine and finally hydrolysis of the imine bond which creates the dication at two of the nitrogen atoms in the quinoxaline ring system.

In the structure of the title bromide salt, the dication is essentially planar with a longer C10—N3 distance of 1.45 (3) Å, compared to the usual C_aro_—N_amine_ single bond distance of 1.43 (3) Å. This might be due to the electron withdrawing effect of positively charged pyridine, which increased the *C*-N_amine_ bond order. Other C—C and C—N bond distances are well within the limits expected for aromatic rings (Koner & Ray, 2008; Kanderal *et al.*, 2005; Fritsky *et al.*, 2006).

The asymmetric unit contains a discrete 2-azaniumyl-3-bromo-6-oxo-5,6-dihydropyrido[1,2-*a*]quinoxalin-11-ium cation, with a protonated amine, pyridine group, and two bromide anion (Fig 1). In title compound, the ions are connected into a three dimensional hydrogen-bonded network *via* N—H···Br and C—H···Br hydrogen bonds (Table 1). All H_ammonium_ atoms and H_pyrazine_ N—H group are involved in hydrogen bonds with two different bromide ions, and each anion accepts hydrogen bonds from three different cations. No intermolecular π–π interations are evident in the hydrocarbon layer in title compound.

### S2. Experimental

Molecular bromine (220 mg, 72.0 mL, 1.40 mmol) was added to a methanolic solution (10 ml) of Schiff base, *N1*,*N4*-bis (pyridine-2-ylmethylene)benzene-1,4-diamine (BPYBD) (197 mg, 0.70 mmol). The color of the solution was immediately changed from yellow to orange. The reaction mixture was stirred for 4 h at room temperature under hood. The resulting yellow precipitate was recovered by filtration, washed several times with a small portions of acetone and then with diethyl ether to give 200 mg (64%) of 2-azaniumyl-3-bromo-6-oxo-5,6-dihydropyrido [1,2-*a*]quinoxalin-11-ium bromide (ABODQ). The crystal of the title compound suitable for X-ray analysis was obtained within 3 days by slow evaporation of the methanol solvent.

### S3. Refinement

H atoms were placed in calculated positions and treated as riding on their parent atoms with C—H = 0.95 Å, N—H = 0.88 or 0.91 Å. U_iso_(H) = 1.5U_eq_(N) for the amino-H atoms and 1.2U_eq_(C,N) for the others.

### Crystal data

**Table:**

C_12_H_10_BrN_3_O^2+^·2Br^−^	*F*(000) = 864
*M**_r_* = 451.96	*D*_x_ = 2.179 Mg m^−^^3^
Monoclinic, *P*2_1_/*c*	Mo *K*α radiation, λ = 0.71073 Å
Hall symbol: -P 2ybc	Cell parameters from 999 reflections
*a* = 5.6782 (2) Å	θ = 2.6–28.6°
*b* = 11.9822 (4) Å	µ = 8.78 mm^−^^1^
*c* = 20.2528 (7) Å	*T* = 100 K
β = 90.891 (2)°	Block, yellow
*V* = 1377.78 (8) Å^3^	0.30 × 0.25 × 0.20 mm
*Z* = 4	

### Data collection

**Table:**

Bruker SMART APEX CCD diffractometer	2424 independent reflections
Radiation source: fine-focus sealed tube	1804 reflections with *I* > 2σ(*I*)
Graphite monochromator	*R*_int_ = 0.106
ω scans	θ_max_ = 25.0°, θ_min_ = 2.0°
Absorption correction: multi-scan (*SADABS*; Bruker, 2001)	*h* = −6→6
*T*_min_ = 0.178, *T*_max_ = 0.273	*k* = −14→14
14369 measured reflections	*l* = −23→24

### Refinement

**Table:**

Refinement on *F*^2^	Primary atom site location: structure-invariant direct methods
Least-squares matrix: full	Secondary atom site location: difference Fourier map
*R*[*F*^2^ > 2σ(*F*^2^)] = 0.047	Hydrogen site location: inferred from neighbouring sites
*wR*(*F*^2^) = 0.130	H-atom parameters constrained
*S* = 0.97	*w* = 1/[σ^2^(*F*_o_^2^) + (0.0801*P*)^2^] where *P* = (*F*_o_^2^ + 2*F*_c_^2^)/3
2424 reflections	(Δ/σ)_max_ < 0.001
173 parameters	Δρ_max_ = 1.23 e Å^−^^3^
0 restraints	Δρ_min_ = −0.88 e Å^−^^3^

### Special details

**Table:**

Geometry. All esds (except the esd in the dihedral angle between two l.s. planes) are estimated using the full covariance matrix. The cell esds are taken into account individually in the estimation of esds in distances, angles and torsion angles; correlations between esds in cell parameters are only used when they are defined by crystal symmetry. An approximate (isotropic) treatment of cell esds is used for estimating esds involving l.s. planes.
Refinement. Refinement of F^2^ against ALL reflections. The weighted R-factor wR and goodness of fit S are based on F^2^, conventional R-factors R are based on F, with F set to zero for negative F^2^. The threshold expression of F^2^ > 2sigma(F^2^) is used only for calculating R-factors(gt) etc. and is not relevant to the choice of reflections for refinement. R-factors based on F^2^ are statistically about twice as large as those based on F, and R- factors based on ALL data will be even larger.

### Fractional atomic coordinates and isotropic or equivalent isotropic displacement parameters (Å^2^)

**Table:**

	*x*	*y*	*z*	*U*_iso_*/*U*_eq_	
C1	1.1684 (11)	0.3466 (5)	0.6250 (3)	0.0348 (16)	
H1	1.1805	0.3779	0.5821	0.042*	
C2	1.3279 (12)	0.3771 (5)	0.6724 (3)	0.0404 (17)	
H2	1.4479	0.4297	0.6628	0.049*	
C3	1.3135 (12)	0.3313 (6)	0.7341 (3)	0.0432 (18)	
H3	1.4246	0.3511	0.7676	0.052*	
C4	1.1375 (12)	0.2564 (6)	0.7472 (3)	0.0408 (17)	
H4	1.1293	0.2230	0.7896	0.049*	
C5	0.9719 (11)	0.2291 (5)	0.6989 (3)	0.0308 (15)	
C6	0.7750 (11)	0.1557 (5)	0.7166 (3)	0.0327 (15)	
C7	0.6487 (11)	0.1672 (5)	0.6019 (3)	0.0284 (14)	
C8	0.4824 (11)	0.1344 (5)	0.5543 (3)	0.0297 (14)	
H8	0.3582	0.0851	0.5655	0.036*	
C9	0.5012 (11)	0.1750 (5)	0.4905 (3)	0.0287 (14)	
C10	0.6803 (11)	0.2482 (5)	0.4745 (3)	0.0272 (14)	
C11	0.8407 (11)	0.2812 (5)	0.5212 (3)	0.0294 (14)	
H11	0.9619	0.3323	0.5104	0.035*	
C12	0.8249 (11)	0.2387 (5)	0.5857 (3)	0.0252 (14)	
N1	0.9939 (8)	0.2736 (4)	0.6368 (2)	0.0262 (11)	
N2	0.6287 (9)	0.1254 (4)	0.6658 (2)	0.0319 (12)	
H2A	0.5160	0.0770	0.6737	0.038*	
N3	0.6987 (9)	0.2912 (4)	0.4080 (2)	0.0343 (13)	
H1N3	0.8278	0.3360	0.4054	0.052*	
H2N3	0.5673	0.3314	0.3976	0.052*	
H3N3	0.7126	0.2334	0.3792	0.052*	
O1	0.7430 (8)	0.1251 (4)	0.7735 (2)	0.0428 (12)	
Br1	0.82448 (12)	0.04974 (5)	0.33038 (3)	0.0399 (2)	
Br2	0.21399 (13)	0.05913 (6)	0.91141 (4)	0.0478 (3)	
Br3	0.26693 (12)	0.12868 (6)	0.42784 (3)	0.0388 (2)	

### Atomic displacement parameters (Å^2^)

**Table:**

	*U*^11^	*U*^22^	*U*^33^	*U*^12^	*U*^13^	*U*^23^
C1	0.035 (4)	0.036 (4)	0.033 (4)	−0.004 (3)	0.000 (3)	0.003 (3)
C2	0.038 (4)	0.036 (4)	0.047 (4)	−0.002 (3)	−0.002 (3)	−0.002 (3)
C3	0.038 (5)	0.053 (5)	0.038 (4)	0.003 (4)	−0.005 (3)	−0.015 (4)
C4	0.039 (4)	0.053 (4)	0.030 (4)	0.001 (3)	−0.002 (3)	−0.007 (3)
C5	0.031 (4)	0.040 (4)	0.021 (3)	0.004 (3)	0.002 (3)	−0.005 (3)
C6	0.030 (4)	0.040 (4)	0.028 (4)	0.006 (3)	−0.002 (3)	−0.008 (3)
C7	0.036 (4)	0.031 (4)	0.018 (3)	0.002 (3)	0.002 (3)	−0.001 (3)
C8	0.026 (4)	0.031 (3)	0.032 (4)	0.001 (3)	0.004 (3)	0.000 (3)
C9	0.029 (4)	0.033 (3)	0.024 (3)	0.004 (3)	−0.008 (3)	−0.005 (3)
C10	0.030 (4)	0.035 (4)	0.016 (3)	0.005 (3)	0.006 (2)	−0.002 (3)
C11	0.023 (4)	0.035 (4)	0.030 (3)	−0.002 (3)	0.007 (3)	−0.002 (3)
C12	0.026 (4)	0.027 (3)	0.022 (3)	0.000 (3)	0.003 (3)	−0.002 (3)
N1	0.026 (3)	0.030 (3)	0.023 (3)	0.001 (2)	0.001 (2)	−0.002 (2)
N2	0.033 (3)	0.037 (3)	0.026 (3)	−0.002 (2)	0.005 (2)	0.002 (2)
N3	0.041 (4)	0.043 (3)	0.020 (3)	0.005 (2)	0.004 (2)	0.002 (2)
O1	0.050 (3)	0.062 (3)	0.016 (2)	−0.007 (2)	0.008 (2)	0.004 (2)
Br1	0.0317 (5)	0.0464 (5)	0.0415 (4)	−0.0044 (3)	0.0032 (3)	−0.0017 (3)
Br2	0.0423 (5)	0.0435 (5)	0.0579 (5)	−0.0028 (3)	0.0115 (4)	−0.0072 (3)
Br3	0.0370 (5)	0.0460 (5)	0.0333 (4)	0.0011 (3)	−0.0044 (3)	−0.0048 (3)

### Geometric parameters (Å, º)

**Table:**

C1—N1	1.346 (7)	C7—N2	1.394 (7)
C1—C2	1.359 (9)	C8—C9	1.387 (8)
C1—H1	0.9500	C8—H8	0.9500
C2—C3	1.368 (9)	C9—C10	1.385 (9)
C2—H2	0.9500	C9—Br3	1.907 (6)
C3—C4	1.372 (10)	C10—C11	1.362 (8)
C3—H3	0.9500	C10—N3	1.449 (7)
C4—C5	1.384 (8)	C11—C12	1.405 (8)
C4—H4	0.9500	C11—H11	0.9500
C5—N1	1.374 (7)	C12—N1	1.462 (7)
C5—C6	1.471 (9)	N2—H2A	0.8800
C6—O1	1.225 (7)	N3—H1N3	0.9100
C6—N2	1.361 (8)	N3—H2N3	0.9100
C7—C12	1.361 (8)	N3—H3N3	0.9100
C7—C8	1.395 (8)		
			
N1—C1—C2	122.3 (6)	C8—C9—C10	120.5 (6)
N1—C1—H1	118.9	C8—C9—Br3	117.1 (5)
C2—C1—H1	118.9	C10—C9—Br3	122.4 (4)
C1—C2—C3	119.2 (7)	C11—C10—C9	120.5 (5)
C1—C2—H2	120.4	C11—C10—N3	119.0 (6)
C3—C2—H2	120.4	C9—C10—N3	120.5 (5)
C2—C3—C4	119.6 (6)	C10—C11—C12	119.2 (6)
C2—C3—H3	120.2	C10—C11—H11	120.4
C4—C3—H3	120.2	C12—C11—H11	120.4
C3—C4—C5	120.4 (6)	C7—C12—C11	120.7 (6)
C3—C4—H4	119.8	C7—C12—N1	119.1 (5)
C5—C4—H4	119.8	C11—C12—N1	120.2 (5)
N1—C5—C4	119.0 (6)	C1—N1—C5	119.5 (5)
N1—C5—C6	122.3 (5)	C1—N1—C12	122.5 (5)
C4—C5—C6	118.7 (6)	C5—N1—C12	118.0 (5)
O1—C6—N2	122.2 (6)	C6—N2—C7	123.2 (5)
O1—C6—C5	122.1 (6)	C6—N2—H2A	118.4
N2—C6—C5	115.7 (5)	C7—N2—H2A	118.4
C12—C7—C8	120.2 (5)	C10—N3—H1N3	109.5
C12—C7—N2	121.4 (5)	C10—N3—H2N3	109.5
C8—C7—N2	118.5 (6)	H1N3—N3—H2N3	109.5
C9—C8—C7	119.0 (6)	C10—N3—H3N3	109.5
C9—C8—H8	120.5	H1N3—N3—H3N3	109.5
C7—C8—H8	120.5	H2N3—N3—H3N3	109.5
			
N1—C1—C2—C3	0.9 (10)	N2—C7—C12—C11	−179.2 (5)
C1—C2—C3—C4	−0.7 (11)	C8—C7—C12—N1	178.6 (5)
C2—C3—C4—C5	−1.5 (11)	N2—C7—C12—N1	−1.1 (9)
C3—C4—C5—N1	3.4 (10)	C10—C11—C12—C7	−1.4 (9)
C3—C4—C5—C6	−175.0 (6)	C10—C11—C12—N1	−179.5 (5)
N1—C5—C6—O1	−172.7 (6)	C2—C1—N1—C5	1.1 (9)
C4—C5—C6—O1	5.7 (9)	C2—C1—N1—C12	−178.7 (6)
N1—C5—C6—N2	6.4 (8)	C4—C5—N1—C1	−3.2 (9)
C4—C5—C6—N2	−175.2 (6)	C6—C5—N1—C1	175.2 (5)
C12—C7—C8—C9	0.6 (9)	C4—C5—N1—C12	176.5 (5)
N2—C7—C8—C9	−179.7 (5)	C6—C5—N1—C12	−5.1 (8)
C7—C8—C9—C10	−0.9 (9)	C7—C12—N1—C1	−178.0 (6)
C7—C8—C9—Br3	−179.4 (4)	C11—C12—N1—C1	0.1 (9)
C8—C9—C10—C11	0.1 (9)	C7—C12—N1—C5	2.3 (8)
Br3—C9—C10—C11	178.4 (4)	C11—C12—N1—C5	−179.6 (5)
C8—C9—C10—N3	−179.5 (5)	O1—C6—N2—C7	174.0 (6)
Br3—C9—C10—N3	−1.1 (8)	C5—C6—N2—C7	−5.1 (8)
C9—C10—C11—C12	1.1 (9)	C12—C7—N2—C6	2.7 (9)
N3—C10—C11—C12	−179.3 (5)	C8—C7—N2—C6	−177.0 (5)
C8—C7—C12—C11	0.6 (9)		

### Hydrogen-bond geometry (Å, º)

**Table:**

*D*—H···*A*	*D*—H	H···*A*	*D*···*A*	*D*—H···*A*
N2—H2*A*···Br1^i^	0.88	2.46	3.322 (5)	167
N3—H1*N*3···Br2^ii^	0.91	2.53	3.432 (5)	171
N3—H2*N*3···Br2^iii^	0.91	2.42	3.287 (5)	160
N3—H3*N*3···Br1	0.91	2.50	3.374 (5)	162
C2—H2···Br2^iv^	0.95	2.91	3.813 (6)	160
C3—H3···Br1^v^	0.95	2.85	3.752 (7)	160
C8—H8···Br1^i^	0.95	2.86	3.672 (6)	144
C11—H11···Br2^ii^	0.95	2.80	3.639 (6)	148

Symmetry codes: (i) −*x*+1, −*y*, −*z*+1; (ii) *x*+1, −*y*+1/2, *z*−1/2; (iii) *x*, −*y*+1/2, *z*−1/2; (iv) −*x*+2, *y*+1/2, −*z*+3/2; (v) *x*+1, −*y*+1/2, *z*+1/2.
