# Supplementary figures and images for: Crystal structure of 2-aza­niumyl-3-bromo-6-oxo-5,6-di­hydro­pyrido[1,2-a]quinoxalin-11-ium dibromide
Source: Acta Crystallogr E Crystallogr Commun. 2015 Jan 1;71(Pt 1):o17–8. doi: 10.1107/S2056989014026127 (PMC4331889; doi:10.1107/S2056989014026127)

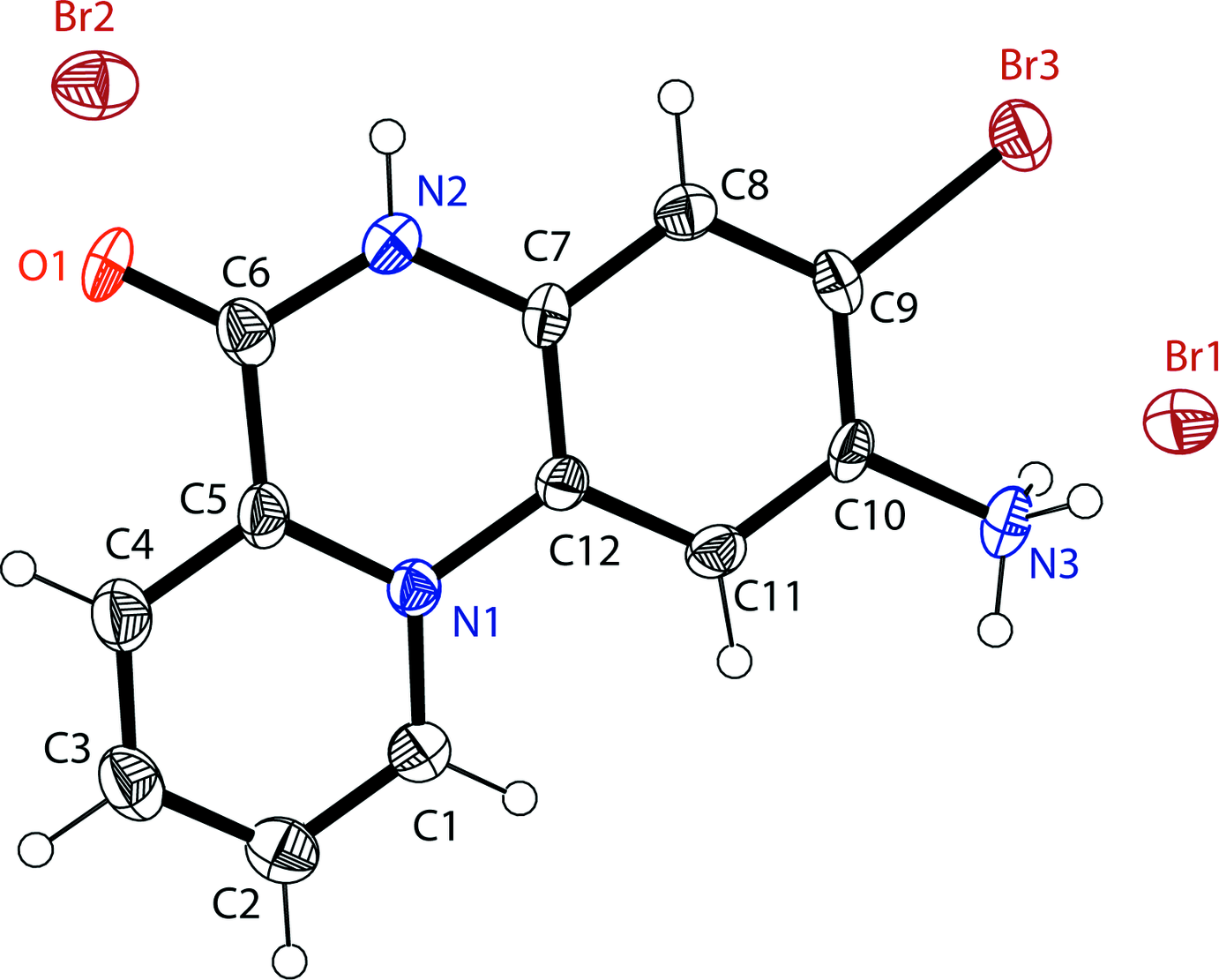

Supplement: Supplementary file 4 [file e-71-00o17-fig1.tif]
